#  An Ex-Vivo Study on the Shaping Parameters of Two Nickel-Titanium Rotary Systems Compared with Hand Instruments

**Published:** 2011-05-15

**Authors:** Maryam Ehsani, Samir Zahedpasha, Ali Akbar Moghadamnia, Jaber Mirjani

**Affiliations:** 1 *Department of Endodontics. Dental school, Babol University of Medical Sciences, Babol, Iran*; 2 *Department of Pharmacology Dental school, Babol University of Medical Sciences, Babol, Iran*; 3 *Dental student, Babol University of Medical Sciences, Babol, Iran*

**Keywords:** Centering Ratio, K-Flexofile, Mtwo, Nickel-Titanium, Race, Shaping Ability

## Abstract

**INTRODUCTION:** Rotary nickel-titanium (NiTi) instruments are thought to allow shaping of narrow, curved root canals more efficiently and more effectively than stainless steel hand instruments. However, the continued search for even more effective and safer instruments has resulted in new rotary systems being introduced on a regular basis. The aim of this study was to compare shaping parameters of RaCe and Mtwo NiTi rotary files with stainless steel K-Flexofile hand instrument.

**MATERIALS AND METHODS:** A total of 60 mandibular first molars with 20-40 degree of curvature in mesial root were divided in to three groups and each was prepared with one kind of instrument (RaCe, Mtwo, stainless steel K-Flexofile). Using pre and post-radiographs, canal curvature was measured, with the Schneider technique. Preparation time was recorded. For evaluating canal centering and transportation, the tooth was sectioned 3, 6 and 9 mm from the apex. Pre and post- preparation photographs were taken from mesiolingual canal. Data was statistically analyzed using One-way ANOVA and Chi-Square tests.

**RESULTS:** RaCe and Mtwo maintained canal curvature better than K-Flexofile (P<0.001). Mtwo prepared the canal in a shorter time (P<0.001).

**CONCLUSION:** Significant statistical difference was not found in the three canal sections between the various systems. RaCe resulted in significantly fewer canal aberrations and better centering ability.

## INTRODUCTION

**T**he goal of root canal preparation is to attain an incessantly tapered canal shape. The smallest diameter should be at the apical foramen and the largest at the canal orifice to allow effective irrigation and obturation (1), without deviations from the original path (2). Moreover techniques and instruments which have the least amount of errors, greatest exactness and the shortest working time (3) should be utilized. Recently developed nickel-titanium (NiTi) files characterized by unique design properties are believed to reduce the incidence of fractures, canal aberrations and the number of procedural steps (4); they produce a funnel-shaped root canal form with great speed and effectiveness (5), maintain the working length (6), respect the original canal shape and therefore remain more centered (7). Since the introduction of these instruments, different NiTi rotary systems have been introduced to the market.

Mtwo (VDW, Munich, Germany) instruments have two cutting edges which form long, almost vertical spirals, ensuring better control of instrument progression throughout the canal.

The posterior aspect of the cutting edges are sharp to optimize cutting efficiency and facilitate advancement of this instrument in the canal. These instruments should be used in a single length technique.

**Figure 1:**
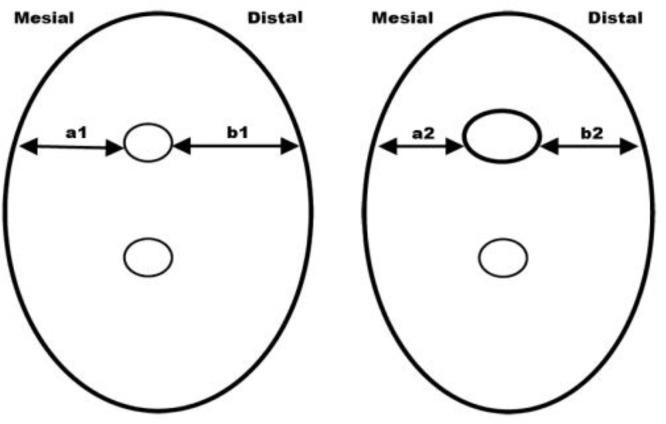
Schematic view of pre- and post-operative cross-section of mesiolingual canal, describing parameters used in Gambill method

RaCe (FKG Dentaire, La Chaux-de-Fonds, Switzerland) rotary instruments have a triangular cross sectional design and alternating cutting edges, a design that is claimed to perform two functions: to eliminate screwing in and blocking in continuous rotation and to reduce the working torque. The RaCe instruments possess a non-cutting tip and are used in a crown-down technique.

K-Flexofile (Dentsply Maillefer, Ballaigues, Switzerland), is made from high-grade stainless steel and twisted triangular cross section to maximize fracture resistance. Outstanding flexibility and cutting efficiency enhanced with a non-cutting tip make them the first choice for curved and narrow canals. The objective of this ex vivo study was to compare the shaping parameters of these two rotary files with stainless steel K-Flexofile hand instrument in molar teeth.

## MATERIALS AND METHODS

A total of sixty freshly extracted human mandibular first molar teeth were selected. Radiographs were taken to evaluate the mesial roots. Double curved and calcified canals were excluded from the study. As assessed by Schneider’s method (8) mesial roots with curvatures of 20^°^ to 40^°^ were included in the study. A muffle-block was constructed, consisting of a u-formed middle section and two lateral walls that were fixed together with three screws. Grooves in the walls of the muffle-block allowed removal and exact repositioning of the complete tooth block or sectioned parts of the tooth. A modification of a radiographic platform, as described by previous researchers, could be adjusted to the outsides of the middle part of the muffle (9,10) (Figure 1). This allowed the exposure of radiographs under standardized conditions. Coronal access cavities were prepared using diamond burs, and the presence of the two separate mesial canals was confirmed by placement of size 15 K-file (Dentsply Maillefer). All the samples were radiographed using periapical Kodak Insight films (Eastman Kodak Company, Rochester, NY) and the radiographic exposure time was 0.8 seconds. Curvature of the mesiobuccal canals was determined by Schneider technique (8). After the preoperative radiograph, the specimens were randomly divided into the following three groups:

***Group 1:*** Mtwo (.04 taper and #10; .05/15, .06/20, .06/25, .05/30 .04/35) enlarged according to the single-length technique.

***Group 2:*** RaCe (.10/40, .08/35, .06/30, .04/25, .02/25, .02/30, .02/35) enlarged according to the crown-down technique.

***Group 3:*** The canal was enlarged sequentially to accept a size 35 K-Flexofile at working length. The taper of the canals was then refined by stepping back in 0.5mm intervals with a larger file size until size 35 K-Flexofile was reached. 

All canals were prepared by a single experienced operator. NiTi files were applied with a 8:1 reduction handpiece (Type 5059; Nouvag, Goldach, Switzerland) powered by a torque-limited endodontic motor (Endo-Mate DT; NSK, Tokyo, Japan) using the recommended torque. Copious irrigation with 1% NaOCl was used throughout the preparation and patency was maintained in all the canals by recapitulation using a K-file size #08. After preparation, standardized radiographs were taken in the same previous position using the muffle with a K-file size #35. Curvatures of the prepared canals were computed using Schneider technique, and were compared with the previous ones. One blind examiner evaluate the specimens root curvatures.

***Preparation time***

Only active instrumentation of the canals was recorded in seconds. This time was computed and recorded by chronometer in all systems. Instrument changes, application of lubricant and irrigation time were not included.

**Table 1:** Mean preparation times (second) and SD with different instruments

**Instruments**	**Mean±SD**	**Number**
RaCe	246.6**±**43.8	20
M-two	202.9**±**7.12	18
K-Flexofile	431.5**±**92.9	17

***Instrument Failure***

Instruments were examined after every use. Deformed or fractured instrument were noted and then replaced.

***Canal Cross Section***

Mesial roots were cut in 3, 6 and 9mm distance from apex by electric saw (Beijing TheLongSuper Technology & Trade Co, China) with 0.3mm diameter according previous study (11). Photograph of mesiolingual canal was provided with digital camera (Sony DSC-S30 cyber shot) under standard conditions before preparation and stored in JPEG format (12). The blocks were again placed in the muffle. Preparation of mesiolingual canal was carried out and photograph was then taken from canals under the same conditions. Sections of prepared root canal were divided into three groups including round, oval and irregular according to previous study (13). Only the irregular sections were considered as unacceptable preparation.

***Evaluation of canal transportation***

The amount of canal transportation was determined by measuring the shortest distance from the edge of uninstrumented canal to the periphery of the root (mesial and distal) and then comparing this with the same measurements obtained from the instrumented images (14) (Figure 1). The following formula was used for the calculation of transportation at each level for both groups: ***(a***_1_***-a***_2_***)-(b***_1_***-b***_2_***), ***Where *a*_1_ is the shortest distance from the mesial edge of the curved root to the mesial edge of the uninstrumented canal; *b*_1_ is the shortest distance from distal (furcation) edge of the curved root to the distal edge of the uninstrumented canal; *a*_2_ is the shortest distance from the mesial edge of the curved root to the mesial edge of the instrumented canal; and *b*_2_ is the shortest distance from distal (furcation) edge of the curved root to the distal edge of the instrumented canal. According to this formula, a result of ″0″ indicates no canal transportation. A result other than ″0″ means that transportation has occurred in the canal.

***Evaluation of centering ability***

According to Gambill *et al.* "the mean centering ratio" indicates the ability of the instrument to stay centered in the canal (14). This ratio was calculated for both the groups at each level using the following ratio: **(*a***_1_**-*a***_2_**) ÷ (*b***_1_**-*b***_2_**)or(*b***_1_**-*b***_2_**) ÷ (*a***_1_**-*a***_2_**)**

If these numbers are not equal, the lower figure is considered the numerator of the ratio. According to this formula, a result of ″1″ indicates perfect centering. 

For the statistical analysis, the data were analyzed using SPSS software version 11.5. One-way ANOVA and Chi-Square test were used. P-values less than 0.05 were considered statistically significant.

## RESULTS

***Preparation Time***

The mean time taken to prepare the canals with different instruments is shown in Table 1. The shortest mean preparation time was recorded with Mtwo instruments (P<0.001).

***Root Canal Curvature Changes (Canal Straightening)***

Average of root canal curvature before preparation was not statistically different among three groups (P>0.05). Following preparation, the most straightening was seen in K-Flexofile group (9.1^º^±3.1^º^). But the difference between the mean straightening of RaCe and Mtwo was not significant (4.9^º^±2.1^º^ and 5.6^º^±1.6^º^, respectively). The difference was statistically significant between hand and rotary files (P<0.001) (Table2).

***Canal Cross Section***

The results concerning post-operative cross-sections of the root canals are given in Table 3. The diameters of the root canals were classified as round, oval, and irregular. Although RaCe achieved the lowest numbers of irregular cross-sections in the middle and coronal third and Mtwo in the apical third, significant statistical difference was not found in any three canal sections between these systems (P>0.05).

**Table 2:** Mean degree of straightening of curved canals and SD after canal preparation with different instruments

**Straightening ** ^(º)^				
**Instruments**	**Mean**	**SD**	**Min**	**Max**
**RaCe**	4.9	2.1	2	9
**Mtwo **	5.6	1.6	2	9
**K-Flexofile**	9.1	3.1	4	15

**Table 3:** Evaluation of postoperative cross-section

**Root canal part**	**Section**	**RaCe**	**M-two**	**K-Flexofile**
Coronal	Irregular	1	5	5
	aAcceptable	19	15	15
Medial	Irregular	3	4	7
	Acceptable	17	16	13
Apical	Irregular	2	1	3
	Acceptable	18	19	17

a :Acceptable cross-sections includes round and oval shapes.

***Root Canal Transportation***

The results are summarized in Table 4. There was no statistically significant difference among three groups in the coronal section (P>0.05). In the apical and middle region the use of RaCe resulted in significantly fewer canal aberrations than Mtwo and K-Flexofile.

***Mean Centering Ratio***

In the coronal part, the difference among three groups was not statistically significant (P>0.05). In the middle and apical part, canals prepared with RaCe instruments remained more centered compared with those enlarged with Mtwo and K-Flexofile (Table 5).

## DISCUSSION

In the present study we evaluated the canal preparation using two rotary system (Mtwo and RaCe) and hand K-Flexofile on natural human teeth. The parameters assessed were preparation time, root canal curvature changes, canal cross section, canal transportation and centering ratio. Human teeth were chosen as they simulate clinical conditions better than acrylic blocks.

**Table 4:** Means±SD of transportation (mm) at different levels

**Instruments (n)**	**Coronal**	**Middle**	**Apical**
RaCe(20)	0.13±0.13	0.06±0.05	0.07±0.05
Mtwo (20)	0.14±0.11	0.10±0.14	0.11±0.05
K-Flexofile (20)	0.12±0.07	0.14±0.14	0.13±0.06
*P-value*	0.846	0.03	0.004

**Table 5:** Centering ratio of instrumentation groups (Mean±SD)

**Instruments (n)**	**Coronal**	**Middle**	**Apical**
RaCe (20)	0.60±0.25	0.74±0.22	0.64±0.22
Mtwo (20)	0.57±0.27	0.50±0.19	0.41±0.25
K-Flexofile (20)	0.56±0.21	0.55±0.22	0.39±0.27
*P-value*	0.881	0.009	0.05

Acrylic resin is not an optimum material to reproduce the microhardness of testing rotary instruments because it does not emulate dentin or the anatomic variations (enlargements, oval root canals, etc.) (15). It has been mentioned that shape of the flutes of NiTi files was altered when used in plastic blocks, which was not seen with natural teeth (16); moreover, rotary instrument will generate heat when used inside the resin block, which will soften the resin material (17). Other studies have shown that the softening of the resin block will lead to binding of cutting blades and increased chance of instrument fracture (18).

The mean preparation time was recorded in seconds by chronometer, which only included the active instrumentation time. Mtwo instruments achieved the shortest mean preparation time was recorded when Mtwo instruments were used (19). This may be because of the S-shaped cross-sectional design of the Mtwo files, resulting in very aggressive cutting edges and positive rake angle, which is known to require less energy to cut dentin than blades with a neutral or negative rake angle.

In the present study, two Mtwo files fractured during canal preparation, but in other two groups, file fracture did not occur. On the other hand, defects in K-Flexofile were more common than the NiTi rotary files. Fracture of NiTi file usually occurs unexpectedly. Less fracture occurrence in the RaCe group can be related to crown-down technique that prevents extra force on the file (20). The higher incidence of fractures of Mtwo files seems to be related to the screw-in effect of these instruments when used according to the single-length technique in S-shaped canals because the whole length of the instrument is subjected to stress, and therefore increased risk of the instrument becoming blocked in a longer canal segment leading to torsional fractures (20).

Total results of canal curvature evaluation indicate that RaCe caused the least canal curvature change although Mtwo had very similar results. Most curvature change occurred in K-Flexofile group. One study compared RaCe with ProTaper and established that RaCe maintained the original curvature perceptibly better than ProTaper (21). Another study demonstrated that Mtwo instruments respected curved canals better than K3 or RaCe instruments (22).

One of the most important requirements of root canal preparation is the complete preparation of the canal. The evaluation of the post-operative cross-sectional area of canals can be used to score shaping ability, since this aspect varies amongst different instruments and techniques (23). All three kinds of files used in this study shaped the canal cross section similarly and they left only a few unacceptable forms. In the coronal part, RaCe did better than Mtwo and K-Flexofile indicating better ability of files with high tapering (10%) in the coronal part. In the middle part of canal, RaCe and Mtwo files (3 and 4 cases of unacceptable form respectively) performed better than K-Flexofile. In the apical part of canal, there was no significant difference. But it seems that Mtwo prepared canal more constantly in this area, because it used different numbers of files frequently in the apical part of canal. Although, there was no similar study in comparison of Mtwo, RaCe and K-Flexofile files, previous studies (24) have not revealed obvious difference between NiTi rotary files and stainless steel hand files.

NiTi rotary instruments maintain canal initial shape in the curved canals better than hand files (25). In the middle and apical parts of canals shaped by RaCe system, transportation occurred less frequently than the two other systems. The crown-down technique may make access for subsequent files easier and more logical. Mtwo performed better than K-Flexofile. This can be related to the higher flexibility of NiTi alloy compared to stainless steel files.

An instrument that remains centered reduces the risk of transportation, zips, elbows, or other mishaps (26). In this study, RaCe had superior centering ratio than the other two files, specially in the middle and apical parts. Moreover, safe and non-cutting tip allows instrument to move in the canal properly and remain central within the canal (27). Flexibility of NiTi instruments can explain this property. Studies on NiTi instruments have shown their better centering ratio than stainless steel hand files (28). Javaheri *et al.* compared Hero 642, RaCe, and Pro taper in canal transportation and found that Pro taper caused more transportation in apical area (29).They suggested that this file be implemented in combination with other less tapered more flexible systems, like RaCe, in preparation of curved canals. In another study RaCe instruments prepared curved root canals with preparation diameters larger than those normally used with minimal canal transportation (30). The results of the present study confirm the results of previous studies on rotary NiTi systems. Overall, the final shapes of canal cross-section were acceptable with few aberrations among the three groups.

## CONCLUSION

In this study, the Mtwo rotary instruments prepared the canals considerably quicker than the other systems. RaCe and Mtwo caused the least canal curvature change.
